# Rückfallprävention bipolarer Störungen: ein clusteranalytischer Ansatz bei einer randomisierten, kontrollierten Psychotherapiestudie

**DOI:** 10.1007/s00115-024-01720-7

**Published:** 2024-08-22

**Authors:** Thomas J. Stamm, J. Fiebig, G. O. Malley, L. M. Sondergeld, P. Treu, F. Bermpohl, E. Friedel, G. Hindi-Attar, E. Quinlivian, S. Schreiter, A. Wietzke, T. Ethofer, A. Fallgatter, I. Lang, E. Beck, K. Krisch, I. Kunze, R. Niebler, J. Zwick, V. Kraft, M. Lambert, Rohenkohl, F. Rühl, M. Bauer, J. Conell, L. Jurjanz, D. Ritter, P. Ritter, M. Spreer, S. Biere, N. Goldbach, S. Kittel-Schneider, S. Matura, V. Oertel, A. Reif, I. Falkenberg, T. Kircher, I. Kluge, S. Mehl, E. Poll, K. Adorjan, M. Heilbronner, J. Kálmán, F. Klöhn-Saghatolislam, T. G. Schulze, F. Senner, O. Gruber, L. Heß, S. Trost, C. Werner, S. Wolter, M. Hautzinger

**Affiliations:** 1 Professur für Klinische Psychiatrie und Psychotherapie, Medizinische Hochschule Brandenburg – Theodor Fontane, Fehrbelliner Straße 38, 16816 Neuruppin, Deutschland; 2 Klinik für Psychiatrie und Psychotherapie CCM der Charité - Universitätsmedizin Berlin, Charitéplatz 1, 10117 Berlin, Deutschland; 3 Klinik für Psychiatrie, Eberhard Karls Universität Tübingen, Geschwister-Scholl-Platz, 72074 Tübingen, Deutschland; 4 Deutsches Zentrum für Psychische Gesundheit (DZPG), Eberhard Karls Universität Tübingen, Geschwister-Scholl-Platz, 72074 Tübingen, Deutschland; 5 Fachbereich Psychologie, Abteilung für Psychologie und Psychotherapie, Eberhard Karls Universität Tübingen, Schleichstr. 4, 72076 Tübingen, Deutschland; 6 Klinik und Poliklinik für Psychiatrie und Psychotherapie, Fachbereich Psychologie, Zentrum für Psychosoziale Medizin, Universitätsklinikum Hamburg-Eppendorf, Martinistraße 52, 20251 Hamburg, Deutschland; 7 Klinik für Psychiatrie und Psychotherapie, Fakultät für Medizin, Universitätsklinikum Carl Gustav Carus, TU Technische Universität Dresden, Fetscherstraße 74, 01307 Dresden, Deutschland; 8 Klinik für Psychiatrie, Psychosomatik und Psychotherapie, Universitätsklinikum Frankfurt – Goethe-Universität, Theodor-Stern-Kai 7, 60596 Frankfurt am Main, Deutschland; 9 Klinik für Psychiatrie und Psychotherapie, Universitätsklinikum Marburg, Baldingerstraße, 35043 Marburg, Deutschland; 10 Klinik für Psychiatrie und Psychotherapie, Institut für Psychiatrische Phänomik und Genomik IPPG, Nußbaumstraße 7, 80336 München, Deutschland; 11 Klinik für Psychiatrie und Psychotherapie, Georg-August-Universität Göttingen, Wilhelmsplatz 1, 37073 Göttingen, Deutschland; 12 Klinik für Gerontopsychiatrie, Universitäre Altersmedizin FELIX PLATTER, Burgfelderstrasse 101, 4055 Basel, Schweiz

**Keywords:** Bipolar 1 Störung, Bipolar 2 Störung, Kognitive Verhaltenstherapie, Unterstützende Psychotherapie, Rückfallprophylaxe, Bipolar 1 disorder, Bipolar 2 disorder, Cognitive behavior therapy, Supportive psychotherapy, Relapse prevention

## Abstract

Anliegen dieser Arbeit ist es, mittels explorativer Auswertungen verschiedene Verlaufstypen bipolarer Störungen und den damit verbundenen Merkmalen zu differenzieren und dabei insbesondere die Rolle von Bipolar-1- und Bipolar-2-Störung sowie den Einfluss der rezidivprophylaktischen Interventionen im Rahmen einer randomisierten, kontrollierten Therapiestudie zu klären. Es konnten 274 der ursprünglich 305 eingeschlossenen Personen untersucht werden. Patienten nahmen entweder an einer kognitiv-verhaltenstherapeutischen (SEKT) oder unterstützenden, patientenzentrierten (FEST) Gruppentherapie teil, welche sich über 4 Therapietage (entspricht 16 Doppelstunden) in monatlichem Abstand erstreckte. Depressive und manische Symptomatik wurde anhand des LIFE jeweils für die zurückliegenden 6 Monate bezogen auf jede Woche vor und nach der Interventionsphase sowie 6 und 12 Monate katamnestisch beurteilt. Die Effekte beider Gruppentherapien erwiesen sich als vergleichbar. Statistisch signifikant wurden in einem multivariaten Proportional-Hazards-Modell jedoch der Faktor Bipolar 1 und 2 sowie die Interaktion von Therapie mit Bipolar 1 und 2. Insbesondere profitierten Bipolar-2-Patienten von der SEKT-Intervention deutlich schlechter als von FEST. Es ergaben sich 3 Cluster, die Bipolar 1 (SEKT, keine Komorbidität, mehrheitlich keine Rezidive, jüngere Patienten) von Bipolar 2 (FEST, keine Komorbidität, mindestens 1, oft 2 Rezidive, ältere Patienten) und von einer heterogenen Gruppe (beide Interventionen, Komorbidität) trennten. Die Unterscheidung von Bipolar-1- und Bipolar-2-Störung ist wichtig und bislang unzureichend berücksichtigt. Bipolar-2-Störungen zeigen generell einen schlechten Verlauf und sprechen auf eine kognitive Verhaltenstherapie (SEKT) besonders schlecht an. Eine offene, unstrukturierte, unterstützende, patientenzentrierte Psychotherapie (FEST) ist generell wirksam.

**Zusatzmaterial online:**

Zusätzliche Informationen sind in der Online-Version dieses Artikels (10.1007/s00115-024-01720-7) enthalten.

## Abstract (en)

The aim of this study was to differentiate between types of bipolar disorders and the associated features using explorative analysis. The focus was particularly on the role of bipolar 1 and bipolar 2 disorders as well as the influence of prophylactic interventions for relapse in a randomized, controlled treatment study. A total of 274 of the 305 originally included persons could be investigated in the study. Patients participated in either cognitive behavioral group therapy (SEKT) or supportive, patient-centered group therapy (FEST). Treatment took place over 4 days separated by a 1-month interval (equivalent to 16 double hours). Depressive and manic symptoms were assessed using the longitudinal interval follow-up evaluation (LIFE). The symptoms were retrospectively assessed for the previous 6 months, with respect to each week before and after the intervention phase and for 6‑month and 12-month follow-ups. The results show that the effects of both group therapies were comparable; however, there were statistically significant differences in a multivariate proportional hazards model for the factors bipolar 1 and 2 as well as the interaction of therapy with bipolar 1 and 2. In particular, bipolar 2 patients benefited significantly less from the SEKT intervention than from the FEST intervention. There were three clusters identified that separated bipolar 1 (SEKT, no comorbidity, predominantly no recurrences, younger patients), from bipolar 2 (FEST, no comorbidity, at least 1 often 2 recurrences, older patients) and from a heterogeneous group (SEKT and FEST, comorbidity). The distinction between bipolar 1 and bipolar 2 disorder is important and has so far not received sufficient attention. Bipolar 2 disorders generally have a worse course and respond particularly poorly to cognitive behavioral therapy (SEKT). An open, unstructured, supportive, patient-centered psychotherapy (FEST) is generally effective.

## Hintergrund

Bipolare Störungen sind schwere, chronisch verlaufende Erkrankungen mit hoher Rate an (Ko‑)Morbidität und Mortalität [2, 7]. Zahlreiche epidemiologische Studien zeigen, dass zwei Drittel der Betroffenen von den Krankheitsepisoden sich zwar erholen, doch davon die Hälfte innerhalb von 2 Jahren trotz stimmungsstabilisierender Medikation eine erneute Krankheitsepisode erleidet [10, 15]. Nationale und internationale Behandlungsleitlinien, gestützt auf Einzelstudien und Metaanalysen empfehlen statt einer Monotherapie die Kombination von Pharmakotherapie und (rezidivprophylaktischer) Psychotherapie, um dadurch die Einnahme der Medikamente, die Krankheitsbewältigung und das Stressmanagement zu verbessern und über 2 Jahre günstigere Verläufe zu erreichen [3, 5, 12]. Vor allem die kognitive Verhaltenstherapie [11] hat sich dabei bewährt, wenngleich die Ergebnisse nicht eindeutig und durchweg günstig sind [4, 14, 21–23]. Miklowitz et al. [13] identifizierten 20 kontrollierte, randomisierte Therapiestudien mit insgesamt 3863 Patienten mit einer bipolaren Störung, die mit einer Kombination von Pharmakotherapie und Psychotherapie behandelt wurden. Als Kontrollbedingung dienten psychiatrische Standardbehandlung bzw. unterstützende Betreuung. Die manualisierten Psychotherapien (kognitive Verhaltenstherapie, Familientherapie, interpersonale Psychotherapie, psychoedukative Therapie) bewirkten deutlich günstigere Verläufe bezogen auf das Wiederauftreten einer affektiven Episode (Odds Ratio 0,56) als die Kontrollbedingungen.

Bereits vor über 10 Jahren konnten wir [10], allerdings an einer kleinen Stichprobe von 76 Patienten, zeigen, dass zwei aktive, wenngleich unterschiedlich gestaltete Psychotherapien bezogen auf die Rückfallverhinderung vergleichbar wirksam sind.

Zur Überwindung zahlreicher Schwächen und Grenzen der früheren Studien, wurde eine große, multizentrische Studie geplant [19] und erfolgreich umgesetzt [6]. Es kamen zwei Arten von Psychotherapie in einem neuen Format zur Anwendung, die adjuvant zur psychiatrischen Standardbehandlung (Medikation, Beratung) hinsichtlich ihrer Wirkung auf Rückfallverhinderung über 18 Monate untersucht wurden. Wir erwarteten, dass durch eine spezifische, kognitiv-verhaltenstherapeutische, fertigkeitenorientierte Psychotherapie (SEKT) die Rückfallrate, d. h. die Entwicklung einer neuen affektiven Episode im Rahmen einer bipolaren Störung, im Vergleich mit einer unterstützenden, dem Patienten Raum lassenden, auf Emotionen fokussierten Psychotherapie (FEST) signifikant niedriger ausfällt. Dieses primäre Erfolgsmaß wurde durch weitere, sekundäre Erfolgsmaße ergänzt, sodass SEKT verglichen mit FEST über den Untersuchungszeitraum hinweg zu weniger subklinischer Symptomatik, weniger depressiver bzw. manischer Symptomatik und höherem sozialem Funktionsniveau führt. Eine weitere Erwartung bezog sich auf Moderatoren zur Identifikation von Indikatoren, die bereits vor Behandlungsbeginn später erfolgreiche von weniger erfolgreichen Krankheitsverläufen zu unterscheiden erlauben. Die untersuchten Einflussgrößen auf den Remissionsverlauf sind: Art der bipolaren Störung (Bipolar 1 = BP1 bzw. Bipolar 2 = BP2), Episodenanzahl, Erkrankungsalter, Schweregrad der (letzten) Episode, Komorbidität, soziodemographische Faktoren (Alter, Geschlecht), frühere Behandlungen und soziales Funktionsniveau als Prädiktoren des Behandlungserfolgs (Wahrscheinlichkeit eines Rückfalls, Zeitstrecke bis Rückfall).

Anliegen dieser Arbeit ist es, diese ergänzende, explorative Auswertung einer größeren Therapiestudie [7] zu verschiedenen Verlaufstypen bipolarer Störungen und den damit verbundenen Merkmalen zu berichten. Mittels Auswertungen von Symptomverläufen, Survival- und Clusteranalysen, geht es vor allem um die Rolle von Bipolar-1- und Bipolar-2-Störungen sowie den Einfluss der rezidivprophylaktischen Interventionen.

## Methode

An der Therapiestudie (im Zeitraum von 9/2016 bis 3/2021) nahmen insgesamt 305 Personen, die die Einschlusskriterien erfüllten, teil (Tab. 1). Einschlusskriterien waren Personen beiderlei Geschlechts zwischen 18 und 55 Jahren, die mit einer Diagnose einer bipolaren Störung (Bipolar 1 oder Bipolar 2) nach DSM sich gegenwärtig in psychiatrischer Behandlung bei individuell angemessener Medikation nach S3-Leitlinien (in der Regel „mood stabilizer“) befanden. Sie mussten stabil (seit mindestens 4 Wochen) remittiert sein (Fremdbeurteilung mittels QIDS-C ≤ 10, YMRS ≤ 12) und während der zurückliegenden 2 Jahre mindestens eine affektive Episode aufweisen. Von allen Personen musste eine informierte Einwilligung (zu den Untersuchungen sowie zur Behandlung, zu den Nachuntersuchungen sowie der Zufallszuweisung) vorliegen. An allen Studienorten lagen positive Bescheide der lokalen Ethikkommission vor.

**Tab. 1:** Stichprobenmerkmale der Studienpatienten

Merkmale		SEKT	FEST	Total
Stichprobe	*N*	154	151	305
Alter (Jahre)	Median	35	33	34
Geschlecht	Weiblich *N*	74	69	143
	Männlich *N*	80	82	162
Diagnose	Bipolar 1 *N*	99	108	207
	Bipolar 2 *N*	55	43	98*
Frühere Episoden	Median	7	7	7
Komorbidität (weitere Diagnosen)		35 %	28 %	32 %
Ausbildung (Schule)	≥12 Jahre	55 %	62 %	59 %
	10–11 Jahre	37 %	33 %	35 %
	≤9 Jahre	7 %	5 %	6 %
Berufsausbildung	Ja	89 %	85 %	87 %
	Nein	11 %	15 %	13 %
Berufstätigkeit	Keine, berentet	38 %	32 %	35 %
	Beschäftigt (≥ 50 %)	51 %	50 %	50 %
	Ausbildung, Minijob	12 %	18 %	15 %
Krankheitsmonate (letzte 5 Jahren – Median)		8	7	8
Jahreseinkommen	> 36.000 €	25 %	22 %	24 %
	24.000–35.000	13 %	16 %	15 %
	12.000–23.000	30 %	25 %	27 %
	< 12.000	32 %	37 %	35 %
Psychiatrische Behandlung, auf Medikation		93 %	89 %	91 %
Lithiummedikation		53 %	56 %	54 %
Zurückliegende stationäre Behandlung		87 %	84 %	85 %
Frühere (ambulante) Psychotherapie		72 %	66 %	69 %

*Verteilung Bipolar 1 und 2 (χ^2^ 17,98; *p* ≤ 0,01) signifikante Unterschiede zwischen SEKT und FEST

**Abb. 1:**
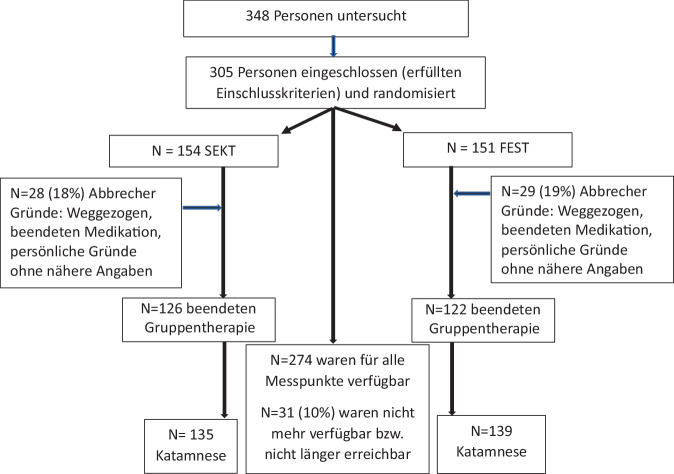
Eingeschlossene (*N* = 305) und vollständig untersuchte (*N* = 274) Teilnehmer

Die Abb. 1 stellt als Flussdiagramm die Zuweisung, die Aufteilung, die Untersuchungen und die Verluste der Studienteilnehmer dar. Von den überwiesenen und grob untersuchten (gescreenten) Personen erfüllten 305 die Einschlusskriterien verbunden mit der Bereitschaft über 18 Monate an der Studie teilzunehmen. Bis zur 1‑Jahres-Katamnese konnten 274 der ursprünglich eingeschlossenen Personen untersucht werden. Für lediglich 31 (10 %) Personen fehlt ein kompletter Datensatz. Während der Gruppentherapien brachen zwar 57 Personen (19 %) die Teilnahme ab, doch waren davon 26 trotzdem bereit, an den Abschluss- und Katamneseuntersuchungen teilzunehmen.

**Abb. 2:**
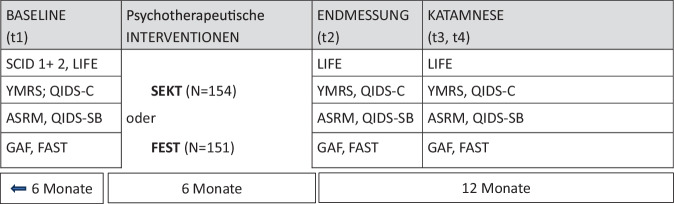
Design, Instrumente und Untersuchungsablauf. Das LIFE [8] deckt jeweils 6 Monate mit wöchentlichen Beurteilungen (PSR-Urteile) retrospektiv ab. Da zur Baseline die zurückliegenden, vor Einschluss in die Studie beurteilten Wochen einfließen, wird ein Gesamtzeitraum von 24 Monaten abgebildet. Weitere, hier nicht berücksichtigte Instrumente sind ASRM [1], GAF/FAST [16], QIDS [17], YMRS [24]. Ergebnisse dazu finden sich in [6]

Der Untersuchungsplan (Abb. 2) sah vor, dass die Teilnehmer nach der Eingangsuntersuchung einer von zwei Gruppentherapien zugewiesen wurden und über 5 Monate in monatlichem Abstand behandelt wurden. Nach der Enduntersuchung erfolgten noch zwei Katamnesetermine nach 6 und 12 Monaten.

Unabhängige, gegenüber den Behandlungen verblindete, speziell und zentral trainierte, in den Zentren angestellte Psychiater bzw. Klinische Psychologen mit mindestens 3 Jahren Berufserfahrung untersuchten die Teilnehmer vor und nach den Interventionen sowie zur Katamnese. Zur diagnostischen Beurteilung wurde das Strukturierte Klinische Interview (SCID), doch auch die klinischen Vorbefunde verwendet. Depressive, manische und hypomanische Symptomatik wurde anhand des Longitudinal Follow-up Evaluation (LIFE, [8]) jeweils für die zurückliegenden 6 Monate beurteilt. LIFE Present State Ratings (PSR-Werte von 1 bis 6 für jede Untersuchungswoche) sind das Haupterfolgskriterium und werden hier zur Verlaufs- und Clusteranalyse herangezogen. Beurteilungen von 5 oder 6 über eine (Manie) bzw. 2 Wochen (Depression) werden als eine neue Krankheitsepisode gewertet. Das LIFE hat sich wiederholt, so auch bei uns, als zuverlässiges Maß für die Beurteilung depressiver und manischer Symptomatik in Wochenintervallen erwiesen (Übereinstimmung der Beurteiler-Kappa 0,78–0,89).

Alle Studienteilnehmer waren in psychiatrischer Behandlung. Diese Behandler, meist niedergelassene Psychiater, folgten den S3-Behandlungsleitlinien, was Medikation (Stimmungsstabilisierer), Kontrolle der Medikamenteneinnahme, mögliche Medikamentenanpassung, erforderliche Untersuchungen, Abklärung von Suizidalität und kurze Beratung umfasste. Zu dieser „treatment as usual“ erhielten alle eine 24 h umfassende Gruppentherapie verteilt über 5 Monate. Die Gruppentherapien fanden im Abstand von 4 bis 6 Wochen jeweils über einen ganzen Tag (von 9 bis 17 Uhr) statt. Dieser Therapietag war unterteilt in 4 Sitzungen zu jeweils 90–100 min mit Pausen dazwischen. Die Teilnehmer erhielten eine von zwei Psychotherapien, beide mit dem Fokus auf Rückfallprophylaxe und Umgehen mit beginnender Symptomatik. Das besondere neue Format (eintägige Gruppentherapien) kam sehr gut an und ermöglichte vor allem entfernter lebende Patienten an den rückfallprophylaktischen Programmen teilzunehmen. Bei einer Anfahrt von über 1 h sind wöchentliche Gruppentherapietermine über einen längeren Zeitraum kaum machbar und mit vielen Fehlzeiten von Teilnehmern verbunden. Längere Anfahrtszeiten alle 4 bis 6 Wochen werden und wurden von Teilnehmern eher akzeptiert.

Die SEKT-Bedingung (siehe elektronisches Zusatzmaterial) ist eine strukturierte, erweiterte kognitive Verhaltenstherapie [11], die aus 5 Modulen besteht und einem Studienmanual folgt. Das allgemeine basale zu Beginn jedes Therapietages angewandte Modul umfasste Achtsamkeitsübungen und (Auswertung von) Selbstbeobachtungen zu Stimmung, Aktivitäten, sozialen Kontakten, Schlaf, Medikation. Es gab dann ein Modul mit Schwerpunkt auf Psychoedukation, Erklärung und Verlauf der Erkrankung, Zusammenhang von Alltagsbelastungen, Stress und depressiver bzw. manischer Symptomatik, Gestaltung einer stabilen Alltagsstruktur, Schlaf-Wach-Rhythmik und Lebensbalance. Ein weiteres Modul drehte sich um Frühwarnzeichen, persönliche Risikofaktoren, Stressmanagement, Problemlösen und interpersonale Fertigkeiten. Ein Modul fokussierte auf kognitive Prozesse und deren Veränderung, dysfunktionales Denken und Informationsverarbeitung, Grübeln, Schuld und Scham. Ein anderes Modul drehte sich um Emotionen, Emotionsregulation, Impulskontrolle und Fertigkeiten zur Überwindung heftiger Emotionen. Alle Module umfassten Arbeitsmaterialien, Übungen, Hausaufgaben. Jedes dieser Module ging, nach dem allgemeinen basalen Modul, über einen ganzen Gruppentherapietag. Teilnehmer von SEKT sollten Verständnis und Fertigkeiten erlernen, um das Auftreten neuer Krankheitsepisoden zu verhindern bzw. abzumildern.

Die FEST-Bedingung (siehe elektronisches Zusatzmaterial) ist eine offene, dem Patienten Raum lassende, supportive Psychotherapie (orientiert an Markowitz [9]). Die Bedürfnisse und Wünsche der Teilnehmer stehen im Vordergrund. Die Gruppentherapeuten nutzen keine Materialien, Übungen oder vorgeplante Strukturen für einen Therapietag. Die Teilnehmer werden ermuntert, aktuelle und persönliche Themen einzubringen, persönliche Erfahrungen, Krisen, Probleme, Gefühle zu nennen und die Reaktionen der anderen Teilnehmer zu erfahren. Ziel ist es, die Interaktion der Teilnehmer zu fördern und wechselseitige Hilfestellung zu ermöglichen. Die Gruppentherapeuten greifen wenig ein, verbalisieren bestenfalls emotionales Erleben von Teilnehmern, lassen ansonsten die Interaktionen zwischen den Gruppenteilnehmern laufen. Das Ziel von FEST, für das es auch ein Therapeutenmanual gibt, ist es, den Teilnehmern zu ermöglichen, ihr emotionales Erleben auszudrücken, Verhalten und Erleben besser zu verstehen, sich bei der Problembewältigung wechselseitig zu unterstützen und die eigenen Ressourcen zu entdecken.

Alle Studientherapeuten (27 Psychotherapeuten bzw. Psychiater mit mehreren Jahren Berufserfahrung) wurden zentral über mehrere Tage in beiden Studieninterventionen trainiert. Um als Studientherapeut akzeptiert zu werden, musste die Einhaltung der Vorgaben durch die Leitung von Therapiegruppen nach FEST bzw. nach SEKT demonstriert und von der Studienleitung akzeptiert werden. Alle Gruppentherapien wurden videografiert und monatlich supervidiert. Es fanden außerdem zentral jährliche Auffrischungs- und Trainingskurse statt. Die Einhaltung der Interventionsprotokolle wurde anhand von 117 (60 SEKT, 57 FEST) jeweils 90-minütigen Videomitschnitten durch unabhängige Beurteiler unter Verwendung eines Beurteilungssystems überprüft und mit guter Übereinstimmung als hoch eingestuft (siehe elektronisches Zusatzmaterial).

Die Zuweisung zu einer der beiden Interventionen erfolgte zentrumsbezogen. Pro Studienzentrum wurde eine Randomisierungsliste erstellt, die 94 potenzielle Zuweisung zu einer der beiden Studientherapien enthielt. Die Randomisierungslisten, erstellt von einem unabhängigen Biostatistiker, waren allein der zentralen Studienleitung (M.H.) zugänglich. Sollte ein passender Teilnehmer an einem Zentrum eingeschlossen werden, dann wurde die Studienleitung informiert, die dann entsprechend der Randomisierungsliste des jeweiligen Studienorts die Zuweisung vornahm und an die lokale Studienleitung zurückmeldete. Das Randomisierungsprotokoll sicherte, dass beide Interventionen an allen Zentren vorkamen und umgesetzt wurden.

**Abb. 3:**
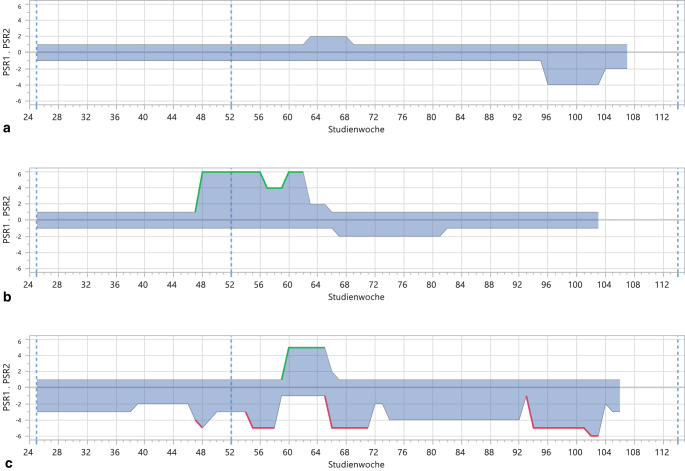
Verlaufsbeispiele anhand LIFE-PSR-Beurteilungen über Untersuchungszeitraum. PSR1 bildet depressive Symptomatik, PSR2 bildet manische Symptomatik ab. PSR-Werte von 5 und 6 entsprechen dem Vollbild einer depressiven bzw. manischen Episode. **a** *Verlauf* *0:* kein Rezidiv im Untersuchungszeitraum (55,4 %). **b***Verlauf* *1:* ein Rezidiv im Untersuchungszeitraum (22,9 %). **c*** Verlauf* *2:* Zwei oder mehr Rezidive im Untersuchungszeitraum (21,7 %). (In den Klammern finden sich die Anteile an den Verlaufstypen in der Untersuchungsstichprobe)

Grundlage für die ergänzende, explorative Verlaufsauswertung bilden die wöchentlichen Beurteilungen im LIFE (PSR 1 bis 6) der komplett dokumentierten Studienteilnehmer. Über einen Zeitraum von 2 Jahren (6 Monate vor Einschluss, 6 Monate während der Intervention, 12 Monate während der Katamnese) wurden wöchentlich Beurteilungen zum Vorliegen depressiver und manischer Symptomatik durch unabhängige Kliniker, speziell in der Anwendung des LIFE trainiert, retrospektiv vorgenommen. Der PSR-Wert von 5 bzw. 6 erfüllt die diagnostischen Kriterien für eine ernsthafte Krankheitsepisode. Die Kriterien einer depressiven Episode werden erfüllt, wenn PSR 5 oder 6 über zwei zusammenhängenden Wochen vergeben wird. Eine manische Episode liegt vor, wenn über eine Woche das PSR 5 oder 6 für manische Symptome vergeben wird. Die Abb. 3 gibt einen Eindruck der dokumentierten Verläufe über den Untersuchungszeitraum. Es lassen sich zumindest 3 Verlaufsgruppen unterscheiden: 0 = keine erneute Krankheitsepisode, 1 = ein affektives Rezidiv, 2 = zwei und mehr affektive Rezidive.

Es wurden ferner „Hierarchical-latent-class“-Clusteranalysen (jmp statistical package 2022) unter Einbezug der Variablen Alter (≤ 34, ≥ 35 Jahre), Geschlecht (m, w), Komorbidität (ja/nein weitere diagnostizierte psychische Störungen), Episodentyp (depressiv, manisch, gemischt), Eingangsdiagnose (Bipolar 1, Bipolar 2), Episodenanzahl vor Studieneintritt (< 7, ≥ 7), Episodenanzahl während Studienzeit (0, 1, ≥ 2) für 2 bis 5 Clusterlösungen berechnet. Clusteranalysen stellen eine statistische Methode dar, um Daten in Gruppen (hierarisch) zu organisieren, basierend auf deren Nähe bzw. Assoziation. Clusteranalysen helfen, Zusammenhänge zwischen Variablen zu verdeutlichen und Hypothesen zu generieren.

## Ergebnisse

**Abb. 4:**
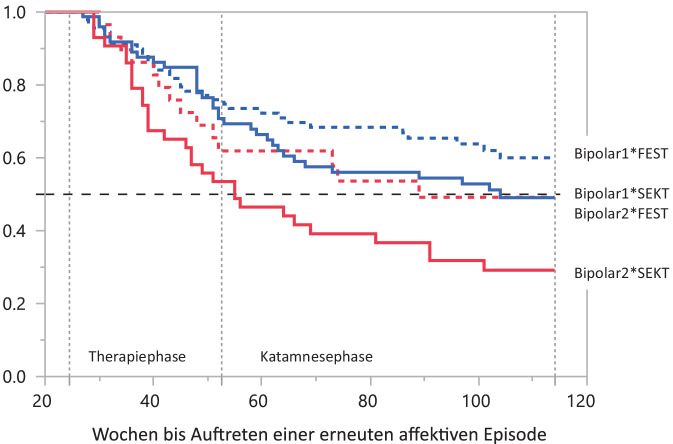
Kaplan-Meier-Survival-Kurven für Bipolar 1 und 2 unter Berücksichtigung der erhaltenen Intervention zur Rückfallprophylaxe (*Sternchen*: statistische Interaktion). Bipolar-I-Patienten haben günstigere Verläufe als Bipolar-2-Patienten. Diese profitieren wenig von einer kognitiven Verhaltenstherapie (SEKT) (Log-Rank-Test χ^2^ = 10,4591, *p* < 0,0150)

Die Effekte beider Gruppentherapien erwiesen sich als vergleichbar (Log-Rank-Test χ^2^ = 0,2827, *p* ≤ 0,5950). Statistisch signifikant wurden in einem multivariaten Proportional-Hazards-Modell [7] jedoch der Faktor Bipolar 1 und 2 sowie die Interaktion von Therapie mit Bipolar 1 und 2 (*p* ≤ 0,01624 und *p* ≤ 0,03118). Kaplan-Meyer-Survival-Kurven mit dem Auftreten einer erneuten affektiven Episode machen deutlich, dass Bipolar-1- und Bipolar-2-Patienten auf die beiden Interventionen zur Rückfallprophylaxe unterschiedlich ansprechen (Abb. 4). Im Studienzeitraum erlitten 45 % der Bipolar-1-Patienten, doch 62 % der Bipolar-2-Patienten ein Rezidiv (neue Erkrankungsepisode). Bipolar-2-Patienten profitierten wenig von SEKT (Rezidivrate 70 %), eher von FEST (Rezidivrate 51 %). Insgesamt erwies sich die unterstützende, patientenzentrierte, unstrukturierte Gruppentherapie (FEST) sowohl bei Bipolar-1- (Rezidivrate 40 %) als auch bei Bipolar-2-Patienten (Rezidivrate 51 %) als hilfreich. Prädiktoren des Behandlungsergebnis zugunsten von SEKT waren komorbid vorliegende Erkrankungen (*p* ≤ 0,02227) und die An- bzw. Abwesenheit bei den Gruppentherapien, was insbesondere die männlichen Teilnehmer betraf (*p* ≤ 0,00110).

**Abb. 5:**
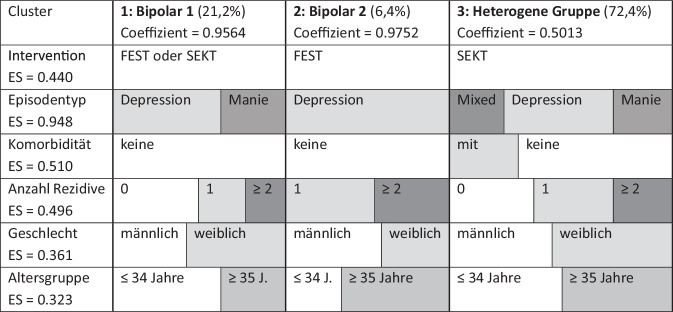
Grafische Darstellung der Ergebnisse der (Latent-class‑)Clusteranalyse

Diese Ergebnisse bilden sich auch in der explorativen Clusteranalyse ab. Insbesondere unterscheiden sich Teilnehmer mit einer Bipolar-1- bzw. Bipolar-2-Störungen hinsichtlich der Rezidive im Verlauf, während Komorbidität eher bei heterogenen Verläufen relevant wird. Es lassen sich 3 interpretierbare Cluster (Loglikelihood-Parameter 783,30) anhand der erhaltenen Gruppentherapie (Effekt 0,440), der Verläufe (Effekt 0,496), dem bipolaren Störungstyp (Effekte 0,07–0,411), der Art affektiver Episoden (Effekt 0,948), der Komorbidität (Effekt 0,510), dem Geschlecht (Effekt 0361) und der Altersgruppe (Effekt 0,323) unterscheiden (Abb. 5).

Das Cluster 1 wird durch Bipolar-1-Störungen ohne Komorbidität gebildet. Im Verlauf kommen sowohl depressive wie manische Episoden vor. Dabei hat die Mehrzahl dieser hier zugeordneten meist jüngeren Patienten im Verlauf kein Rezidiv. Beide Therapieverfahren erbringen vergleichbare rückfallprophylaktische Ergebnisse.

Das Cluster 2 wird durch Bipolar-2-Störungen ohne Komorbidität repräsentiert. Diese kleine, meist ältere Gruppe hat im Untersuchungszeitraum meistens eine, doch oft zwei und mehr depressive Episoden. Die unstrukturierte, unterstützende Intervention (FEST) wirkt hier am ehesten rückfallverhindernd.

Das größte, heterogene Cluster 3 umfasst sowohl Bipolar-1- als auch Bipolar-2-Erkrankte. Viele haben komorbide psychische Störungen. Im Verlauf kommen depressive, manische und gemischte Episoden vor. Hier erweist sich SEKT als erfolgreiche rückfallprophylaktische Intervention.

## Diskussion

Entgegen unseren Erwartungen erweist sich eine komplexe, strukturierte kognitive Verhaltenstherapie einer unterstützenden, unstrukturierten, dem Patienten Raum lassenden Psychotherapie in der Verhinderung von Rezidiven nicht überlegen. Sowohl die multivariate Proportional-Hazards-Modellierung als auch die (Latent-class‑)Clusteranalyse zeigen, dass die Unterscheidung von Bipolar-1- und Bipolar-2-Störung und die Interaktion von einer Bipolar-1- bzw. -2-Störung und der erhaltenen Gruppentherapie Einfluss auf den Erkrankungsverlauf nehmen. Bipolar-2-Störungen zeigen generell einen schlechten Verlauf, was sich in der erhöhten Rezidivrate zeigt. Bipolar-2-Störungen sprechen auf eine KVT (SEKT) sehr viel schlechter an als Bipolar-1-Störungen. Überraschend gut bewährt sich die unterstützende, unstrukturierte Psychotherapie (FEST). Weitere Einfluss- bzw. Clusterbildungskriterien waren komorbide Erkrankungen und die Compliance bei der Gruppentherapie. Patienten mit komorbiden Störungen profitieren mehr (weniger Rezidive) von der KVT (SEKT).

Es liegen bislang keine Ergebnisse vor, die Bipolar-1- und Bipolar-2-Patienten hinsichtlich ihrer Krankheitsverläufe und hinsichtlich ihres Reagierens auf verschiedene Psychotherapien direkt miteinander vergleichen. Es ist uns nur eine Therapiestudie bekannt [20], die gezielt Bipolar-2-Patienten mit einer akuten depressiven Episode in eine Psychotherapie (Interpersonal and Social Rhythm Therapy, IPSRT) im Vergleich mit einer Kombinationstherapie (IPSRT plus Medikation) einschloss. Dort zeigte sich, dass die Kombinationstherapie gegenüber der Psychotherapie bezüglich der Reduktion depressiver Symptomatik und der Verhinderung von Rezidiven während der Katamnese mit geringem, doch signifikantem Effekt überlegen war. Diese Studie zeigt, dass IPSRT auch bei Bipolar-2-Patienten in einer depressiven Phase gut ankommt und wirkungsvoll ist. Lediglich das Ergebnis, dass die Kombinationstherapie (IPSRT + Medikation) sich günstig auf den Verlauf (weniger Rezidive) auswirkt, kann zum Vergleich zu unseren Ergebnissen herangezogen werden. Beide Psychotherapien (SEKT, FEST) zeigen in Kombination mit Medikation und psychiatrischer Betreuung (TAU) bei euthymen Patienten rezidivprophylaktische Effekte. Das unterschiedliche Ansprechen von Bipolar-1- und Bipolar-2-Patienten auf die beiden aktiven Psychotherapien ist neu und unerwartet. Macht jedoch deutlich, dass die Bipolar-2-Gruppe sich von der Bipolar-1-Gruppe unterscheidet, was sich vor allem in der höheren Rezidivrate ausdrückt. Bipolar-2-Patienten bedürfen offensichtlich passendere Interventionen zur Rezidivprophylaxe. Die strukturierte, mit vielen Materialien und Übungen befrachtete kognitive Verhaltenstherapie (SEKT) trifft nicht deren Bedürfnisse, zumal möglicherweise bei dieser Patientengruppe bereits ausreichende Ressourcen vorliegen. Möglicherweise liegt der Fokus bei SEKT zu sehr auf dem Thema Manie, wohingegen die Bipolar-2-Patienten vor allem mit Depressionen und deren Verhinderung zu kämpfen haben. Hier müssen die Therapieangebote angepasst und auf die Bedürfnisse der Bipolar-2-Patienten zugeschnitten werden. Interessant ist, dass unsere offene, unstrukturierte, emotions- und patientenzentrierte Psychotherapie (FEST) dies bereits gut trifft und günstigere Ergebnisse liefert. Ein Grund dafür könnte darin liegen, dass über zwei Drittel unserer Studienpatienten bereits psychotherapieerfahren waren und in den zurückliegenden Jahren an rückfallprophylaktischen Interventionen teilgenommen hatten. Die FEST-Bedingung aktiviert bereits erworbene Ressourcen ohne Altbekanntes zu wiederholen oder Vorschriften zu machen. Zukünftige Studien sollten, je nach Fragestellung, nach Bipolar 1 und Bipolar 2 stratifizieren oder nur die eine bzw. die andere Patientengruppe einschließen. Bei vorliegender Komorbidität sind die Ergebnisse sowohl für Bipolar 1 als auch für Bipolar 2 weniger günstig. Sowohl SEKT als auch FEST berücksichtigt diese Bedingung unzureichend. Auch hierfür sind ergänzende bzw. alternative Interventionen zu entwickeln und zu evaluieren.

Das neue, sich über einen ganzen Tag erstreckende, im Abstand von 4 bis 5 Wochen stattfindende Format der Gruppenpsychotherapie kam sehr gut an. Die Rückmeldungen der Teilnehmer sind positiv und es gelang tatsächlich, auch Patienten mit längeren Anfahrten für die Studienteilnahme zu gewinnen. Die Effekte dieser Gruppenpsychotherapie sind vergleichbar zu früheren Studien und decken sich mit aktuellen Metaanalysen [13]. Es ist uns bewusst, dass unser Psychotherapieformat von allen bislang durchgeführten Therapiestudien und auch der klinischen Praxis abweicht. Wir sehen den Vorteil, nicht nur darin, dass Patienten erreicht werden, die in ländlichen bzw. abgelegenen Gebieten leben, sondern auch in der intensiven Beschäftigung (Übung) mit einzelnen Inhalten sowie der vermehrten Interaktion zwischen den Teilnehmern während der Pausen. Nach unserer Beobachtung kam es zu intensiveren, persönlichen Gesprächen sowie wechselseitiger Unterstützung. Möglicherweise ein weiterer Aspekt, der bei FEST dafür viel mehr Raum lässt und zu deren günstigen Ergebnissen beiträgt. Wenn Patienten Symptomverschlechterung (vor allem depressive Symptomatik) erleben, könnte eine regelmäßigere, wöchentliche Gruppenpsychotherapie hilfreicher sein, um diese Verschlechterung zu bewältigen und ein Rezidiv zu verhindern. Das Thema unterschiedliche Formate von oder gar personalisierter, modularer Psychotherapie (vgl. Schramm et al. [18]) und deren Effekte auf den Interventionserfolg ist unzureichend untersucht.

Kritisch gegen die hier berichtete Untersuchung lässt sich einwenden, dass im Design ein dritter Kontrollarm mit der alleinigen Anwendung von TAU fehlt, was die Bewertung des rezidivprophylaktischen Effekts der beiden Kombinationstherapien (SEKT + TAU, FEST + TAU) erschwert. Wir verzichteten auf ein dreiarmiges Design, da hinreichende Evidenzen aus Vorläuferstudien und Metaanalysen vorliegen, die eine deutliche Überlegenheit von TAU in Kombination mit Psychotherapie gegenüber TAU allein belegen [13, 20]. Ferner erfordert ein dreiarmiges Design eine zumindest zweifach größere Stichprobe, was weder finanziell noch organisatorisch machbar erschien.

Limitierend muss auch gesehen werden, dass wir für die hier berichteten ergänzenden Auswertungen nicht die komplette, sondern eine um Abbrecher bzw. fehlende Datensätze reduzierte Stichprobe zur Verfügung hatten. Inwiefern diese Selektion an kooperativen Teilnehmern auf die Ergebnisse Einfluss nimmt, ist schwer abzuschätzen, muss jedoch als Störgröße bedacht werden.

Ein weiterer kritischer Einwand kann sein, dass die Studientherapeuten beide Therapieformen je nach Randomisierungsergebnis anwenden mussten. Hierdurch kann es zu Problemen mit der „allegiance“ (Loyalität, Überzeugtheit) bzw. zu einem Diffusionseffekt (Angleichung) kommen. Dieses Problem ist nicht endgültig und vollständig zu lösen. Bei getrennten, von der jeweiligen Methode überzeugte Therapeuten liegt hohe „allegiance“ und kein Diffusionseffekt vor, dafür mögen Persönlichkeits- und Interaktionsfaktoren der Therapeuten in den Therapiegruppen unterschiedlich wirken. Wir entschieden uns dafür, den Diffusionseffekt dadurch möglichst geringhalten, indem wir Studientherapeuten umfangreich und wiederholt trainierten sowie über den Studienzeitraum regelmäßig supervidierten. Außerdem führten wir videogestützte, unabhängige Adhärenzbeurteilungen durch (siehe elektronisches Zusatzmaterial), die dafür sprechen, dass es den Studientherapeuten gelang SEKT und FEST sauber und klar zu trennen.

Gegenüber Clusteranalysen wird häufig kritisch eingewandt, dass sie eher der „Kunst als der Wissenschaft“ zuzuordnen sind. Angesichts des sehr großen Clusters 3, kann die Berechtigung für diese explorative Auswertung in Zweifel gezogen werden. Eindeutig dominiert dieses heterogene, durch Bipolar 1, Bipolar 2 und Komorbidität, eher durch SEKT-Intervention geprägte Cluster. Es erschien uns informativ, die beiden kleinen, eher homogenen Cluster zu berichten. Diese unterstreichen die bereits auf anderem Weg gewonnenen Ergebnisse, insbesondere den deutlich ungünstigeren Verlauf der Bipolar-2-Störungen, für die eine kognitive Verhaltenstherapie in der hier durchgeführten Form wenig hilfreich ist. Wir sehen in den explorativen Verlaufs- und Clusteranalysen keine hypothesen- bzw. strukturprüfenden Auswertungen, sondern wollten damit die früheren multivariaten Auswertungen [7] ergänzen, um auf Einflussvariablen, Stratifizierungsmöglichkeiten und Interventionsentwicklungen für zukünftige Untersuchungen hinzuweisen.

## Schlussfolgerung

Sowohl für die psychiatrische als auch für die psychotherapeutische Behandlung erweist sich die Unterscheidung von Bipolar-1- und Bipolar-2-Störung als wichtig und bislang unzureichend berücksichtigt. Bipolar-2-Störungen zeigen generell einen schlechten Verlauf, was sich in der erhöhten Rezidivrate zeigt. Bipolar-2-Störungen sprechen auf eine kognitive Verhaltenstherapie (SEKT) mit dem Fokus auf Rezidivprophylaxe schlecht an. Eine unstrukturierte, unterstützende, dem Patienten Raum lassende Psychotherapie (FEST) erweist sich generell als wirksam. Die Ergebnisse legen eine personalisierte, modularisierte Rezidivprophylaxe nahe, wozu bislang empirische Evidenzen ausstehen.

## Supplementary Information

Im Supplement erfolgt eine Kurzbeschreibung der beiden Psychotherapieformen SEKT und FEST sowie die Ergebnisse der Adhärenzbeurteilungen der Studientherapeuten bezogen auf die Umsetzung bzw. Einhaltung der Therapiemanuale.
