# Erratum zu: Rückfallprävention bipolarer Störungen: ein explorativer, clusteranalytischer Ansatz bei einer randomisierten, kontrollierten Psychotherapiestudie

**DOI:** 10.1007/s00115-025-01853-3

**Published:** 2025-07-11

**Authors:** Thomas J. Stamm, J. Fiebig, G. O. Malley, L. M. Sondergeld, P. Treu, F. Bermpohl, E. Friedel, G. Hindi-Attar, E. Quinlivian, S. Schreiter, A. Wietzke, T. Ethofer, A. Fallgatter, I. Lang, E. Beck, K. Krisch, I. Kunze, R. Niebler, J. Zwick, V. Kraft, M. Lambert, Rohenkohl, F. Rühl, M. Bauer, J. Conell, L. Jurjanz, D. Ritter, P. Ritter, M. Spreer, S. Biere, N. Goldbach, S. Kittel-Schneider, S. Matura, V. Oertel, A. Reif, I. Falkenberg, T. Kircher, I. Kluge, S. Mehl, E. Poll, K. Adorjan, M. Heilbronner, J. Kálmán, F. Klöhn-Saghatolislam, T. G. Schulze, F. Senner, O. Gruber, L. Heß, S. Trost, C. Werner, S. Wolter, M. Hautzinger

**Affiliations:** 1 Professur für Klinische Psychiatrie und Psychotherapie, Medizinische Hochschule Brandenburg – Theodor Fontane, Fehrbelliner Straße 38, 16816 Neuruppin, Deutschland; 2 Klinik für Psychiatrie und Psychotherapie CCM der Charité - Universitätsmedizin Berlin, Charitéplatz 1, 10117 Berlin, Deutschland; 3 Klinik für Psychiatrie, Eberhard Karls Universität Tübingen, Geschwister-Scholl-Platz, 72074 Tübingen, Deutschland; 4 Deutsches Zentrum für Psychische Gesundheit (DZPG), Eberhard Karls Universität Tübingen, Geschwister-Scholl-Platz, 72074 Tübingen, Deutschland; 5 Fachbereich Psychologie, Abteilung für Psychologie und Psychotherapie, Eberhard Karls Universität Tübingen, Schleichstr. 4, 72076 Tübingen, Deutschland; 6 Klinik und Poliklinik für Psychiatrie und Psychotherapie, Fachbereich Psychologie, Zentrum für Psychosoziale Medizin, Universitätsklinikum Hamburg-Eppendorf, Martinistraße 52, 20251 Hamburg, Deutschland; 7 Klinik für Psychiatrie und Psychotherapie, Fakultät für Medizin, Universitätsklinikum Carl Gustav Carus, TU Technische Universität Dresden, Fetscherstraße 74, 01307 Dresden, Deutschland; 8 Klinik für Psychiatrie, Psychosomatik und Psychotherapie, Universitätsklinikum Frankfurt – Goethe-Universität, Theodor-Stern-Kai 7, 60596 Frankfurt am Main, Deutschland; 9 Klinik für Psychiatrie und Psychotherapie, Universitätsklinikum Marburg, Baldingerstraße, 35043 Marburg, Deutschland; 10 Klinik für Psychiatrie und Psychotherapie, Institut für Psychiatrische Phänomik und Genomik IPPG, Nußbaumstraße 7, 80336 München, Deutschland; 11 Klinik für Psychiatrie und Psychotherapie, Georg-August-Universität Göttingen, Wilhelmsplatz 1, 37073 Göttingen, Deutschland; 12 Klinik für Gerontopsychiatrie, Universitäre Altersmedizin FELIX PLATTER, Burgfelderstrasse 101, 4055 Basel, Schweiz

**Erratum zu:**

**Nervenarzt 2024**

10.1007/s00115-024-01720-7

Die Angaben zur Autorenschaft in der ursprünglich online publizierten Version waren unvollständig. Bitte beachten Sie die korrekten und vollständigen Angaben. Die Online-Version wurde korrigiert.

