# Supplementary material for: Relapse prevention of bipolar disorders: a cluster analytical approach in a randomized controlled psychotherapy study
Source: Nervenarzt. 2024 Aug 22;96(5):471–9. [Article in German] doi: 10.1007/s00115-024-01720-7 (PMC12411313; doi:10.1007/s00115-024-01720-7)
Supplement: Supplementary file 1 — Im Supplement erfolgt eine Kurzbeschreibung der beiden Psychotherapieformen SEKT und FEST sowie die Ergebnisse der Adhärenzbeurteilungen der Studientherapeuten bezogen auf die Umsetzung bzw. Einhaltung der Therapiemanuale. [file 115_2024_1720_MOESM1_ESM.docx]

**Studientherapien**

Bei der **SPEZIFISCHEN PSYCHOTHERAPIE (SEKT)** handelt es sich um eine in dieser Form

noch nicht ausreichend untersuchte Therapieform, die gezielt auf Patienten mit einer Bipola-

ren Störung zugeschnitten ist. Es werden bewährte kognitiv-behaviorale Elemente (vgl. Meyer

& Hautzinger 2013) ergänzt um metakognitive und emotionsregulierende Interventionen zur

Rückfallprophylaxe angeboten und mit den Betroffenen trainiert. Wir gehen davon aus, dass

bestimmte Fertigkeiten benötigt werden, um die Instabilität in der Stimmung und im Antrieb,

die alltäglichen Belastungen und Anzeichen beginnender Episoden zu beeinflussen. Über ins-

gesamt 4 ganztägige Therapie-Workshops (Module), verteilt über 4-5 Monate bearbeitet und

übt eine Gruppe von Betroffenen (zwischen 4 und maximal 7 Teilnehmer) unter Anleitung (1-

2 Therapeuten) diese Fertigkeiten, die sich in der klinischen Erfahrung als hilfreich erwiesen

haben. Dazu gehören (1) Aufklärung und Information, (2) Stimmungs- und Aktivitätenbe-

obachtung, (3) Alltagsgestaltung und Schlaf-Wach-Rhythmus, (4) soziale und zwischen-

menschliche Fertigkeiten, (5) kognitive und metakognitive Hilfen, (6) Emotionsregulations-

techniken, (7) Stressregulation und Selbstfürsorge sowie (8) Umgang mit Frühwarnzeichen,

Krisen und Notfällen. Zwischen den ganztägigen Therapieworkshops bekommen die Teilneh-

mer Aufgaben und Übungen. Unter anderem werden sie dazu angehalten, täglich ihre Stim-

mung zu beobachten und diese in einem Stimmungstagebuch (Protokollbogen) schriftlich fest-

zuhalten. Hierzu werden sie ggf. durch die Therapeuten elektronisch (E-Mail, SMS) erinnert.

Aufbau SEKT:

4 Therapietage (jeweils von ca. 9-17 Uhr, ca. monatlicher Abstand), als offene Gruppe mit

Übungen und zahlreichen Materialien. Aufgliederung in ein über alle Sitzungen konstantes

allgemeines Modul sowie einzelne Schwerpunkte pro Sitzung (Module 1 bis 4).

Allgemeines Modul: Achtsamkeit, Selbstbeobachtung und Aufgaben bearbeiten

Zu Beginn jedes Therapietages werden die ersten beiden Stunden darauf verwendet, mit den

Teilnehmern Übungen zur Achtsamkeit durchzuführen, die Übungen und Aufgaben seit der

letzten Therapiesitzung zu besprechen, die Selbstbeobachtungen auszuwerten und ggf. The-

men aus früheren Modulen zu wiederholen und zu vertiefen.

Modul 1: Psychoedukation, Tagesstruktur, Life Balance

Psychoedukation mit Austausch persönlicher Erfahrungen, Vermittlung von Basiswissen zur

Bipolaren Störung, Erklärungsmodell und Wissen zur Medikation, Erarbeitung des persönli-

chen Krankheitsverlaufs („Lifechart“), Zusammenhänge zwischen Ereignissen, Verhalten,

Symptomen erkennen, Alltagsgestaltung, Tagesstruktur, Schlaf-Wach-Rhythmus, Alkohol- und

Suchtmittelkonsum.

Modul 2: Relative Normalität, Frühwarnzeichen, Verhaltensänderungen

Was ist „normal/abnormal“ im Befinden, Denken und Verhalten; Identifikation von Frühwarn-

zeichen und Erarbeitung gegensteuernder Maßnahmen, Aufbau sozialer und kommunikativer

Fertigkeiten durch Rollenspiele und Übungen, Problemlösen zwischenmenschlicher, berufli-

cher, finanzieller oder anderer mit der Erkrankung einhergehender Probleme.

Modul 3: Kognition und Metakognition

Zusammenhang von Denken und Emotionen/Befinden/Verhalten, Erkennen typischer forma-

ler Denkstörungen (Grübeln und Gedankenrasen), Auseinandersetzung mit Denkverzerrungen

(Geistiger Filter, übertriebene Verallgemeinerung, Zuschreibungsstil, Perfektionismus).

Übungen, Realitätstestung, Alternativerklärungen, metakognitive Interventionen.

Modul 4: Emotionswahrnehmung, Emotionsregulation

Nicht-bewertendes Wahrnehmen und Benennen von Gefühlen, Akzeptanz und Toleranz,

effektive Selbstunterstützung in belastenden Situationen, Emotionsregulation mittels kogniti-

ver Neubewertung und Ablenkung.

Metaanalysen zeigen, dass eine Psychotherapie mit den meisten der genannten Elementen

hilfreich, Rückfall verhindernd und Krankheitsepisoden lindernd ist (Mühlig et al. 2013). Es

werden dabei die in den S3-Leitlinien genannten psychotherapeutischen Prinzipien umge-

setzt:

 Psychoedukation, Vorbereitung auf Krisen und Notfälle

 Selbstbeobachtung von Stimmungsveränderungen, Ereignissen, Verhalten, Denken

 Reflexion von Erwartungen und Maßstäben

 Förderung von Kompetenzen zum Selbstmanagement von Stimmungsschwankungen

und Frühwarnzeichen

 Normalisierung und Stabilisierung von Schlaf-Wach- und sozialem Lebens-Rhythmus

 Stressmanagement und Aktivitätsmanagement

 Steigerung der Selbstwirksamkeitsüberzeugung

Neu sind das Format, also die Therapie- und Trainingstage in monatlichem Abstand, sowie der

Einbezug von Emotionsregulation und metakognitiven Strategien

**Supportive, Psychoedukative Psychotherapie (FEST)**

Die informierende, weniger stark strukturierte und nicht gezielt auf Neulernen ausgerichtete,

das Erleben aktivierende, UNTERSTÜTZENDE PSYCHOTHERAPIE (FEST) kann als allgemeine,

weniger spezifische Behandlung bezeichnet werden. FEST (orientiert an dem BSP/SYSP-Ma-

nual – Hautzinger, 2009) unterstützt in erster Linie die Persönlichkeit, die bei jedem vorhan-

denen Ressourcen und die Selbststeuerung (Selbstkontrollpotentiale). Sie beinhaltet die all-

gemeinen Wirkfaktoren psychologischer Interventionen, von denen angenommen wird, dass

sie den Großteil zum Erfolg von Psychotherapie beitragen. Dazu gehören soziale und emotio-

nale Unterstützung, Verstärkung, Klärung von Motivation und Zielen, Aktivierung von Emoti-

onen und eigenen Ressourcen, Information und Wissensaustausch. In insgesamt 4 ganztägi-

gen Workshops (Module), verteilt über 4-5 Monate bearbeiten eine Gruppe von Betroffenen

(4-7 Teilnehmer) unter Anleitung (1-2 Therapeuten) die Erfahrungen mit der Krankheit und

helfen sich wechselseitig, Selbstkontrolle über Symptomatik, Instabilität, Verunsicherung und

Verlauf zu erlangen bzw. zu stärken. Ein wichtiges Ziel ist, dass Patienten die Bipolare Störung

sowie die Medikation besser verstehen und besser mit ihr umgehen können. Der Austausch

über persönliche Erfahrungen mit der Erkrankung und der Medikation sowie die persönlichen

Bewältigungs- und Verarbeitungsstrategien stehen im Mittelpunkt. Die Rolle der Therapeuten

ist weniger strukturierend, als vielmehr helfend, zulassend, gelegentlich beratend, doch vor-

rangig die Interaktion zwischen den Teilnehmern fördernd. Da es Hinweise gibt (Meyer &

Hautzinger 2012), dass unterstützende, allgemeine Psychotherapie bei Bipolaren Patienten

hilfreich ist, wird sie in dieser Untersuchung als bewährte Behandlung und aktive Alternative

zur (neuen, spezifischen, übenden, aufwändigen) SEKT eingesetzt. Als einziges Therapiemate-

rial wird hier ein Text (Ratgeber) zur Psychoedukation (Meyer & Hautzinger 2013) zugelassen

und an alle Studienpatienten ausgehändigt.

Erlaubtes und günstiges Therapeuten-Verhalten:

Aufmerksamkeit auf und Primat der Affekte (emotionales Erleben), positive Wertschätzung

und Empathie, Verbalisierung und Klärung, Konkretisieren von Emotionen, beruhigende, nor-

malisierende und unterstützende Versicherungen, Geduld, Zeit, gewähren lassen,

Aushalten, Patienten bestimmen die Inhalte und den Verlauf der Tage, Zuwendung, Blickkon-

takt, Anerkennung und Lob, Beobachtungen ansprechen, Stärken und Ressourcen hervorhe-

ben, Transparenz, Erklären, Informieren, Störungen und Irritationen ansprechen,

Klären und Konkretisieren, interessiertes Nachfragen mit Bezug auf Affekt, Zusammenfassung

mit Schwerpunkt auf emotionalen Erfahrungen, optimistisch, nie aufgebend, hoffnungsvoll.

Wenn Teilnehmer interagieren, möglichst gewähren lassen.

Aufbau FEST:

4 Therapietage (jeweils von ca. 9 bis 17 Uhr, ca. monatlicher Abstand), als offene Gruppe,

inhaltlich sind alle Tage gleich gestaltet, es existiert lediglich eine optionales Modul Psycho-

edukation, das bei Bedarf eingesetzten werden kann.

10

Optionales Modul Psychoedukation: Verwendung der dazu vorliegenden Materialien, Bezug

zu „Ratgeber“-Text, Elemente von diesem Modul können zu jedem der vier Gruppenthera-

pietage besprochen werden. Es stellt kein eigenständiges Modul für einen ganzen Therapietag

dar, sondern wird stundenweise während den Therapietagen bearbeitet, falls dafür seitens

der Teilnehmer Bedarf geäußert wird. Inhalte können sein: Persönliche Erfahrungen mit Er-

krankung, Life Chart (Krankheitsverlauf), Psychoedukation, Vermittlung von Basiswissen zur

Bipolaren Störung, Erklärung (Ursachen), Wissen zu Medikation, Compliance, Ratgeber lesen.

Dabei wird auch auf die persönlichen Fragen und Anliegen der Teilnehmer eingegangen.

Therapiemodule 1, 2, 3, 4: Austausch über persönliche Erfahrungen mit Krankheit, Erfahrun-

gen und Erlebnisse während den letzten Tagen und Wochen (letzter Monat), konkrete Bei-

spiele der Teilnehmer nutzen, Austausch zu Themen untereinander anregen, Themenvor-

schläge könnten sein: Tagesgestaltung, Schlaf-Wach-Rhythmus, Ungeduld, Impulsivität, Aus-

wirkung der Krankheit auf Familie/Beziehung, Arbeitstätigkeit, andere über Krankheit infor-

mieren.

**Beurteilung der Adhärenz der Studientherapeuten zu Therapieprotokollen**

Ratingbogen zur Einschätzung der A2Bipolife-Studientherapeuten

| 1. Th. ist unterstützend, freundlich, optimistisch. | **0** | **1** | **2** | **3** | **4** | **5** |
| --- | --- | --- | --- | --- | --- | --- |
| 1. Th. modifiziert automatische Gedanken oder Grundüberzeugungen mit Hilfe von kognitiven Techniken. | **0** | **1** | **2** | **3** | **4** | **5** |
| 1. Th. verzichtet auf die Verwendung jeglichen strukturförderlichen Materials. | **0** | **1** | **2** | **3** | **4** | **5** |
| 1. Therapeutin/Therapeut hilft Patienten ihren Alltag/ihre Woche besser zu strukturieren und auf eine angemessene Balance von Tätigkeiten und Schlaf-Wach-Rhythmus zu achten. | **0** | **1** | **2** | **3** | **4** | **5** |
| 1. Th. gelingt zutreffende Verbalisierungen emotionaler Erlebnisinhalte der Patienten. | **0** | **1** | **2** | **3** | **4** | **5** |
| 1. Th. stellt Zusammenhänge zu Themen her, die für Patienten in einer vorherigen Sitzung wichtig gewesen waren bzw. zur Sprache kamen. | **0** | **1** | **2** | **3** | **4** | **5** |
| 1. Th. bietet in unsystematischer Weise Erklärungen, Informationen, an, ohne einem bestimmten Therapiemodell zu folgen und zu dominieren, verweist bestenfalls auf den Ratgeber. | **0** | **1** | **2** | **3** | **4** | **5** |
| 1. Th. bestärkt Patienten darin, ausreichende Ressourcen (Selbstkontrolle) zu besitzen, um den Verlauf ihrer Bipolaren Störung selbst beeinflussen zu können und Rückfälle zu verhindern. | **0** | **1** | **2** | **3** | **4** | **5** |
| 1. Th. spricht Patienten direkt an und bezieht Patienten in das Gruppengeschehen ein. | **0** | **1** | **2** | **3** | **4** | **5** |
| 1. Th. bestärkt (lobt) für Kooperation, Umsetzung oder Erprobung der Modulelemente. | **0** | **1** | **2** | **3** | **4** | **5** |
| 1. Th. geht während der Sitzung bei Äußerungen vor allem auf die Emotionen der Patienten ein. | **0** | **1** | **2** | **3** | **4** | **5** |
| 1. Th. betont die Bedeutung von Denken, Fühlen und Verhalten für die Stimmungsregulation, bietet kognitiv-behaviorale (Re)Formulierungen an. | **0** | **1** | **2** | **3** | **4** | **5** |
| 1. Th. spiegelt die Besorgnisse/Beschwerden der Patienten, um so das Besprechen dieser Sorgen zu erleichtern. | **0** | **1** | **2** | **3** | **4** | **5** |
| 1. Th. vermittelt den Eindruck eines interessierten, nicht-wertenden Zuhörers, der keinem festgelegten Plan folgt, sondern jedes von den Patienten initiierte Gespräch unterstützt. | **0** | **1** | **2** | **3** | **4** | **5** |
| 1. Th. bietet in geplanter Weise Erklärungen und Informationen (Psychoedukation) an, nimmt dabei Bezug auf das multifaktorielle (psychobiologische) Therapiemodell. | **0** | **1** | **2** | **3** | **4** | **5** |
| 1. Th. verhält sich geduldig, unterbricht nicht und gibt Patienten den benötigten Raum und die benötigte Zeit, egal wohin das führt. | **0** | **1** | **2** | **3** | **4** | **5** |
| 1. Th. stellt Zusammenhänge zu Themen früherer Module her (Überblick). | **0** | **1** | **2** | **3** | **4** | **5** |
| 1. Th. ermöglicht Patienten, Inhalt und Verlauf der Sitzung selbst zu bestimmen, gibt keine Struktur vor oder versucht eine einzuhalten. | **0** | **1** | **2** | **3** | **4** | **5** |
| 1. Th. benutzt die Therapie- und Informationsmaterialien, teilt diese an Teilnehmer aus. | **0** | **1** | **2** | **3** | **4** | **5** |
| 1. Th. ist aktiv und strukturiert, erstellt bzw. folgt dem Therapieplan und setzt die anstehenden Module, Übungen um. | **0** | **1** | **2** | **3** | **4** | **5** |
| 1. Th. unterstützt den Austausch von Erfahrungen zwischen den einzelnen Teilnehmern ohne ein Thema vorzugeben bzw. zu dominieren. | **0** | **1** | **2** | **3** | **4** | **5** |
| 1. Th. ermutigt Patienten, neue Verhaltensweisen und Aktivitäten anzuwenden und zu erproben. | **0** | **1** | **2** | **3** | **4** | **5** |
| 1. Th. macht einen sicheren, entspannten, ruhigen, kompetenten Eindruck. | **0** | **1** | **2** | **3** | **4** | **5** |
| 1. Th. bespricht Aufgaben, Übungen bzw. führt diese mit den Patienten durch. | **0** | **1** | **2** | **3** | **4** | **5** |

*Raters*

We employed three master-level psychology students to rate treatment adherence and competence. All of the raters were independent to the A2 study and received approximately 20 h of training in the use of the scale and introduction to SEKT and FEST. The focus of this training was to be able to identify the presence or absence as well as intensity of the specific SEKT and FEST behaviours in a videotaped therapy session.

*Procedures*

Overall, 117 videos of group treatment (n = 60 SEKT sessions, n = 57 FEST session) were assessed by the raters. In order to assess interrater reliability, thirty videos thereof were rated by all three raters. Videotapes of 60-90 minutes length were randomly selected (three videos per study therapist and therapy type). The raters were given the videos and were not informed which videotapes were of which therapy type. Raters watched the videos and completed the rating scale individually. To prevent rater drift all the raters met on occasion to review the ratings of single rated videos.

*Inter-rater Reliability*

The reliability of the ratings obtained from the three raters was examined by calculating intraclasscorrelation coefficients (ICC), treating the raters as random effects (Strout & Fleiss, 1979, Model 2). The reliability for both subscales SEKT and FEST was very high, ICC_(2,1)_ = 0.98, p < .001 for both subscales. The competence subscale demonstrated moderate reliability, ICC_(2,1)_ = 0.70, p < .001.

*Internal Consistency*

To determine whether the rating scale items were consistent with each other, we calculated Cronbachs α. The alpha for the SEKT and FEST subscale was 0.99 each, representing an excellent internal consistency. The competence subscale had a good internal consistency with α = .88.

*Treatment Differences (siehe Abbildung)*

In order to assess treatment adherence we looked for treatment differences between the two therapy types by comparing the mean scores for each treatment group on the SEKT and FEST subscale. Adherence ratings of the SEKT treatment sessions were significantly higher on the SEKT subscale (*M* = 28.44, *SD* = 6.30) than on the FEST subscale (*M* = 8.86, *SD* = 6.39), *t*(59) = 13.65, *p* <.001. In addition, adherence ratings of the SEKT subscale were significantly higher for SEKT treatment compared to FEST treatment (*M* = 3.53, *SD* =2.08), *t*(72.36) = -29.01, *p* <.001. A similar pattern was observed for FEST, with significantly higher adherence ratings for the FEST treatment sessions on the FEST subscale (*M* = 37.57, *SD* =5.63) relative to the SEKT subscale (M = 2,6, SD 1,9), *t*(56) = 40.47, *p* <.001. Furthermore, adherence ratings of the FEST subscale were significantly higher for FEST treatment compared to SEKT treatment, *t*(114.38) = 25.83, *p* <.001.
